# Resistance of the Liver to Putrefaction

**Published:** 1841-09

**Authors:** 


### RESISTANCE OF THE LIVER TO PUTREFACTION.

Dr. Solon Borland of Memphis, Tenn., has communicated to us the following fact: In the spring of the year 1840, Dr. Frayser of that place exhumed the skeleton of an Indian buried in 1836, and found *all* the soft parts decomposed, except the liver, gall-bladder and bile, which were perfectly natural.

